# Synchronous Occurrence of Primary Breast Carcinoma and Primary Colon Adenocarcinoma

**DOI:** 10.1155/2017/7048149

**Published:** 2017-10-25

**Authors:** Gurkan Yetkin, Fevzi Celayir, Ismail Ethem Akgun, Ramazan Ucak

**Affiliations:** ^1^SBU Sisli Hamidiye Etfal Hospital General Surgery Clinic, İstanbul, Turkey; ^2^SBU Sisli Hamidiye Etfal Hospital Pathology Clinic, İstanbul, Turkey

## Abstract

A 65-year-old female patient presented to the emergency clinic with abdominal pain, meteorism, and intermittent rectal bleeding. Colonoscopy was performed, and a hepatic flexure tumor was detected. Histopathological examination of biopsy revealed adenocarcinoma. Thoracoabdominal CT was performed for staging, and a spiculated contour mass was found incidentally on the left breast. Mammography and ultrasonography were performed for the cause of these findings, and suspicious lesions of malignancy were seen in the left breast. Invasive ductal carcinoma was detected in core needle biopsy samples from lesions. In the multidisciplinary council consisting of oncologist, pathologist, radiologist, and general surgery specialist, it was decided to perform breast operation first and then colon operation, followed by adjuvant chemotherapy. In the first operation, left total mastectomy and sentinel lymph node biopsy were performed. One week after her initial operation, the patient underwent right hemicolectomy. After operations, the patient did not develop postoperative complications and was sent to medical oncology department for adjuvant chemotherapy.

## 1. Background

Synchronous primary malignant tumors are difficult to diagnose and plan for treatment. It is possible to skip asymptomatic synchronous tumors at the time of diagnosis. Because of the lack of definitive guidelines for synchronous tumors, evaluation of the clinical findings of the patient and establishment of the patient-specific treatment strategy are necessary. These can be achieved with a multidisciplinary approach. We present a case in which synchronous breast tumor was detected incidentally on a computed tomography for staging of a colon tumor.

## 2. Case Presentation

A 65-year-old female patient presented with complaints of right upper quadrant pain, meteorism, and intermittent rectal bleeding. She stated that her pain and dyspeptic complaints were lasting for 2 months, and for the last 15 days, she had rectal bleeding in small amounts twice. Physical examination showed pain and tenderness with palpation in the lower and upper right quadrants. There was no pathology at the digital rectal examination. There was no characteristic in the history and family history of the patient.

## 3. Investigations

**Figure 1:**
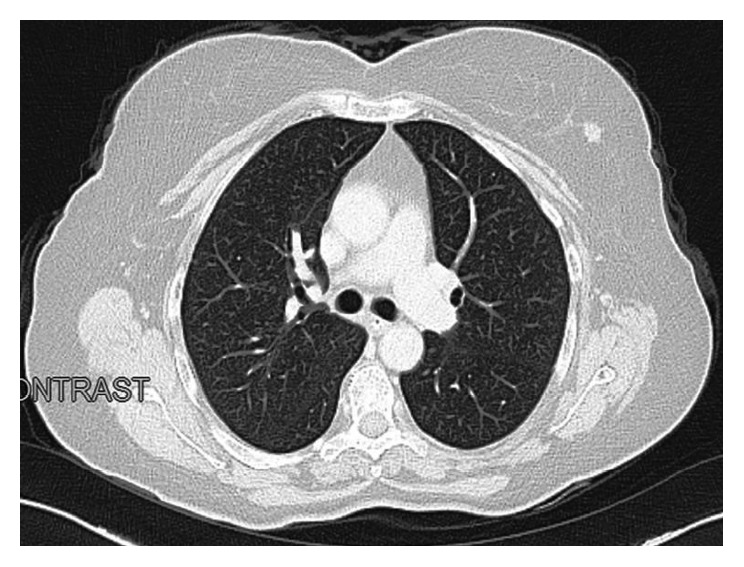
Thoracoabdominal CT.

**Figure 2:**
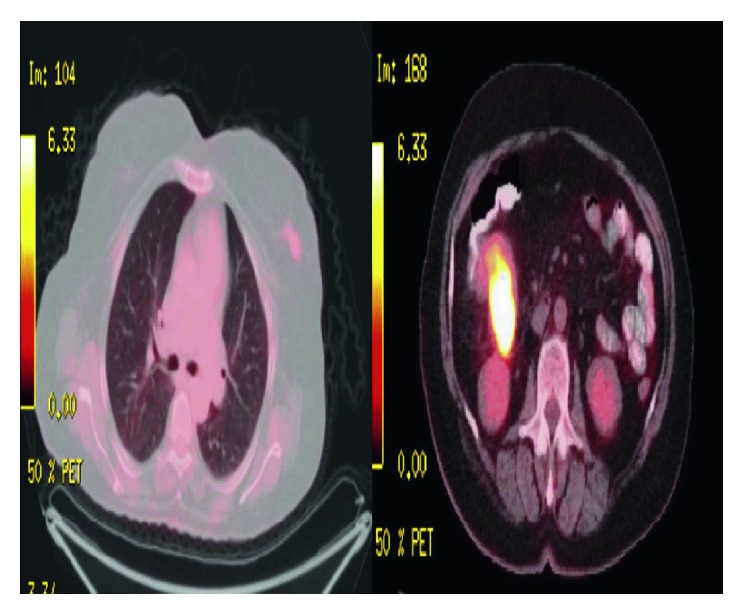
PET-CT showing increased FDG uptake.

Laboratory examinations revealed hypochromic microcytic anemia. The occult blood test in the stool was positive. An ulcerative mass in the hepatic flexure region without obstruction was seen on colonoscopy. Histopathologic examination of the biopsies revealed adenocarcinoma. Thoracoabdominal tomographic examination for the staging of colon cancer revealed multiple thickening of the colonic wall consistent with malignancy, especially in the hepatic flexure level and multiple mesenteric lymphadenomegalies. In addition, a spicule-shaped lesion with massive mass, 2 cm in diameter, was found in the outer quadrant of the left breast (Figure 1()). On a whole-body PET-CT examination, a lesion with increased FDG uptake accompanied by an increase in irregular wall thickness at hepatic flexure level was seen (Figure 2()). The patient's breast was reexamined, and a palpable mass was not detected. On mammogram, a 15 mm diameter hyperdense lesion was observed in the upper external quadrant of the left breast with a spiculated contour. A second focal spot was seen with the same features and 18 mm in diameter near this lesion. Both lesions were confirmed by breast ultrasonography. Invasive ductal carcinoma was detected in ultrasound-guided core needle biopsy of these lesions. So, bifocal breast cancer was detected. Immunohistochemical examination revealed positive estrogen and progesterone receptors. Cerb B2 was negative, and Ki-67 ratio was 5%. We also performed a cytokeratin 7, cytokeratin 20, ER, PR, and HercepTest immunohistochemistry on one colon adenocarcinoma paraffin block to ensure that the colon adenocarcinoma represents a primary malignancy and not metastasis from breast carcinoma. The test result was negative.

## 4. Treatment

In the multidisciplinary oncology council, it was decided to perform breast surgery first. The patient underwent simple mastectomy and sentinel lymph node biopsy. Since no invasion was seen in SLNB in frozen examination, axillary dissection was not performed. There were no complications in the early postoperative period. On the sixth postoperative day, right hemicolectomy and ileotransversostomy were performed. Histopathological examination of the surgical specimens revealed 12 mm and 15 mm diameter invasive ductal carcinoma in two separate foci of the mammary gland. There was no metastasis in SLNB, and the pathological stage was T1N0. Examination of the colon segment revealed an invasive adenocarcinoma of 7 × 6 × 2 cm in size, with invasion to the level of muscularis propria. There were 28 lymph nodes in the mesentery of the resected colonic segment. No metastasis was detected. The pathological stage was identified as T2N0Mx.

## 5. Outcome and Follow-Up

After both surgeries, the patient did not develop postoperative complications and was sent to the medical oncology department for adjuvant chemotherapy.

## 6. Discussion

Cancers developing from multiple origins are called multiple primary cancers (MPCs) and are rarely seen. The average frequency is reported between 0.73% and 11% [1]. If two different tumors originating in the same patient are detected at the same time or within 6 months, this is a synchronous tumor. If the second tumor is detected 6 months later, it is called a metachronous tumor [2]. We detected a synchronous breast cancer incidentally in a tomographic examination for staging in our case with primary colon cancer. The most common malignancy in women is breast cancer. The second one is colon cancer. Fischer et al. reported that the incidence of breast and colon cancer in women at the same time is 3.85% [3]. Various imaging modalities (such as CT, MR, and PET-CT) are used in the staging and monitoring of malignancies. It should always be kept in mind that synchronous tumors may be encountered during their evaluation. As a matter of fact, Karayiannakis et al. [4] had randomly seen synchronous breast cancer in the chest CT scan. Karaduman et al. [5] have detected synchronous colon cancer in patients who have had PET-CT examinations to investigate breast cancer. The clinical and pathological features of synchronous tumors are not fully established. Kimura et al. reported a correlation between familial history and synchronous tumors [6]. Our patient does not fulfill the criteria of cancer family syndrome; therefore, no genetic testing was done to her [7]. There is no definitive guideline for the treatment of synchronous tumors. Because of this, the treatment plan of the patient is specially determined for each patient as the outcome of the multidisciplinary discussions. In our multidisciplinary council consisting of oncologist, pathologist, radiologist, and general surgery specialist, it was decided to perform the operation of breast cancer first and then colon carcinoma, followed by adjuvant chemotherapy. In this decision, no colonic obstruction and bleeding in the patient suggested that emergent surgery was not required for colon carcinoma. The lesser mortality of breast surgery affects our decision also. In fact, the first surgery that is thought to have less morbidity makes it possible to perform the second operation in a short period of time, since it does not disturb the general condition of the patient [8]. In patients with synchronous tumors, the prognosis depends on each tumor stage independently. Synchronous tumors can be said to have no worse prognosis with effective treatment [9].

## 7. Learning Points/Take Home Messages

1. It should be remembered that in patients with malignancy, a second tumor of a different origin should be considered in systemic physical examination and in all laboratory tests and imaging performed.
2. There is no definitive guideline for the treatment of synchronous tumors. The treatment plan should be planned specifically for each patient with a multidisciplinary approach.
3. The prognosis of synchronous tumors depends on the stage of each tumor independently.
